# True infection or contamination in patients with positive *Cutibacterium* blood cultures—a retrospective cohort study

**DOI:** 10.1007/s10096-022-04458-9

**Published:** 2022-05-25

**Authors:** Jolin Boman, Bo Nilson, Torgny Sunnerhagen, Magnus Rasmussen

**Affiliations:** 1 Section for Infection Medicine, Department of Sciences Lund, Lund University, Lund, Sweden; 2 Department of Clinical Microbiology, Office for Medical Services, Region Skåne, Lund, Sweden; 3 Division of Medical Microbiology, Department of Laboratory Medicine Lund, Lund University, Lund, Sweden; 4 Department of Clinical Microbiology, Copenhagen University Hospital, Rigshospitalet, Copenhagen, Denmark; 5 Division for Infectious Diseases, Skåne University Hospital, Lund, Sweden

**Keywords:** *Cutibacterium*, Bacteremia, Contamination, Blood culture

## Abstract

*Cutibacterium* is a genus often considered a contaminant when present in blood cultures, but it can also cause severe infections, especially related to implanted foreign materials. We investigated the incidence and features of patients with true *Cutibacterium* infection. Patients with positive *Cutibacterium* blood cultures between the years 2015–2020 in southern Sweden were identified through microbiology records and medical records were studied retrospectively. *Cutibacterium* isolates were species determined using MALDI-TOF MS. Patients were classified as having true infection or contamination according to a definition considering both clinical and microbiological features and these groups were compared. A total of 313 episodes of positive *Cutibacterium* blood cultures were identified in 312 patients. Of these, 49 (16%, corresponding to an incidence of 6 cases per million inhabitants per year) were classified as true infections. The most common species was *Cutibacterium acnes* (87%), and the majority were elderly men with comorbidities. Patients with true *Cutibacterium* infection often had an unknown focus of infection (*n* = 21) or a focus in the respiratory tract (*n* = 18). We identified one episode of ventriculo-peritoneal shunt infection, three episodes of aortic stent-graft infection, and one episode of infective endocarditis. Two patients, where *Cutibacterium* was isolated at the site of infection, had only one positive blood culture. The finding of positive *Cutibacterium* blood cultures should not always be considered contamination. Definitions of true *Cutibacterium* bacteremia with a demand that more than one blood culture must be positive may miss true infections.

**Supplementary Information:**

The online version contains supplementary material available at 10.1007/s10096-022-04458-9.

## Introduction

*Cutibacterium* is a genus of Gram-positive rods recently established due to the reclassification of the genus *Propionibacterium* [1]. Therefore, all previous studies of *Propionibacterium* will be referred to herein as *Cutibacterium.* The genus consists of several species, including *C. acnes*, *C. avidum*, *C. granulosum*, *C. modesum*, *C. namnetense*, and the most recent, C. *porci* [2, 3]. *Cutibacterium* is often considered a contaminant when isolated from blood cultures and not a pathogen that causes true infections [1, 4]. There is no generally accepted definition of contamination and infection, and therefore authors have used different definitions. This makes it challenging to conclude the incidence of true *Cutibacterium* infections, and the incidence of such infections may therefore be underestimated [5, 6]. There are a few studies performed to distinguish between contamination from the skin and true bloodstream infection with *Cutibacterium.* These studies found a proportion of true infection in patients with positive *Cutibacterium* blood cultures of between 0 and 3.5% [7–10]. Some studies only required that more than one blood culture must be positive for *Cutibacterium* to be considered a true infection, whereas other studies also included a demand on systemic signs of infections and/or signs of localized infection [4, 7, 11, 12]. Previous studies of *Corynebacterium* and coagulase-negative *Staphylococci* (CoNS)*,* bacteria that also often contaminate blood cultures, claimed that it may be reasonable to consider a single positive blood culture as sufficient if a foreign intravascular device was present [13, 14].

*C. acnes* is the most studied species within the genus and is especially known for its involvement in the skin condition of acne vulgaris [15]. *C. acnes* has been recognized to cause infections related to implanted foreign materials and is known to form biofilms on such surfaces [16]. For example, *C. acnes* cause ventriculo-peritoneal (VP) shunt infections, prosthetic joint infections, vascular stent-graft infections, and even infective endocarditis (IE) [11, 17–24]. Although *Cutibacterium* foreign body infections or IE are rare, the diagnosis can be delayed or even missed due to the slow-growing nature of the bacterium, the gradual onset of symptoms, or dismissal of the pathogen as contamination [4, 25, 26]. There are limited studies conducted on species other than *C. acnes* within the genus, but C*. avidum* and *C. granulosum* have, in rare cases, been reported to cause similar infections [27–31].

To the best of our knowledge, there are no studies made to investigate the incidence of true infection in patients with positive blood culture for *Cutibacterium* since the reclassification of the genus or involving the new species within the *Cutibacterium* genus. The aim of this work was therefore to examine the incidence and features of patients with true *Cutibacterium* infection and those with *Cutibacterium* contamination of blood cultures.

## Methods

### Microbiology and species determination

Patients with positive blood cultures for *Cutibacterium* were identified from the registry of the Clinical Microbiology Laboratory, Region Skåne, in Lund in the county of Skåne, Sweden, between the years 2015–2020. The laboratory is the only one in Skåne, a province with a population of 139,0000 inhabitants (December 31, 2020, data from Statistics Sweden (available at https://www.scb.se)), and all cultures from this province (including ten hospitals and all primary care facilities) are handled by this laboratory. The laboratory has satellite blood culture cabinets (BACTEC FX, Becton Dickinson, Franklin Lakes, USA) at the local hospitals, where blood cultures are put into the cabinets at all hours. The blood culture bottles used were BACTEC Plus Aerobic and Lytic Anaerobic. Cultures are normally incubated until positive or for a maximum time of 120 h. Species identification of isolates from positive blood cultures was performed using microflex MALDI-TOF MS (Bruker, Bremen, Germany), with the software flexControl 3.4 and MALDI BioTyper (MBT) Compass 4.1, and the reference database MBT Compass Library DB-8468. First, the Sepsityper kit (Bruker, Billerica, MA) [32] was used in combination with the ethanol-formic acid extraction method for direct and rapid preparation of positive blood cultures for the MALDI-TOF MS analysis. Second, the positive blood cultures were also plated and cultured anaerobically on fastidious anaerobe agar plates (Neogen), and the resulting colonies were prepared by the direct colony method. In cases where a low MALDI BioTyper score was achieved, the ethanol-formic acid extraction method was performed on colonies as described by the instrument manufacturer. The same cut-off value was used as suggested by the instrument manufacturer for a MALDI BioTyper score to be reliable to the species level: ≥ 1.7 and < 2.0 for the genus level and ≥ 2.0 for the species level. In cases where antibiotic resistance identification was made, resistance was determined according to EUCAST protocols, and breakpoints were defined according to EUCAST guidelines at the time of isolation.

### Patients and episodes

Medical records were studied retrospectively. Patients with inaccessible medical records, with blood cultures only obtained from a central venous catheter, and patients under the age of 18 were excluded. The first blood culture with *Cutibacterium* for each patient was considered the index culture for the episode with a positive blood culture. In patients with multiple positive blood cultures, a separate episode of positive *Cutibacterium* blood culture was considered to have happened in cases of a new positive blood culture if at least 7 days of antibiotic treatment had been given, or if at least 30 days had passed between positive cultures. This was based on the protocol used in previous studies on *Enterococcus* [33]. A new episode within 6 months in the patient with the same *Cutibacterium* species was considered a relapse. Data such as age, gender, use of immunosuppressive drugs or chemotherapy, and comorbidities using the updated version of the Charlson comorbidity index were collected [34]. We also collected information if the patient was treated in an intensive care unit, CRP at blood culture, sequential organ failure assessment (SOFA) score for the sepsis-3 classification within 24 h of the blood culture, in-hospital mortality, and death within 30 or 60 days [35]. An episode was defined as a nosocomial infection if signs and symptoms appeared after 48 h or more past hospital admission and health care-associated defined according to Friedman ND et al. [36, 37].

### Defintions

We adjusted the definition of true infection or contamination previously described by Rasmussen et al*.* [14]*,* which in turn was based on the definition by Finkelstein et al.[13]., in patients with blood cultures positive for *Corynebacterium*. Our definition is presented in Table 1. For patients with two or more positive blood cultures for *Cutibacterium*, three criteria had to be fulfilled. Infection had to be confirmed (criterion 1) either by signs of infection (at time of the blood culture, or within 48 h, one of the following: temperature ≥ 38, chills, or leukocytosis > 12 × 10^9^/L) or diagnosed as an infection by the treating physicians. If a patient had a positive blood culture with more pathogenic bacteria (Supplementary Table 1, left column), true infection was rejected (criterion 2). Some bacteria were regarded as having the same degree of pathogenicity as *Cutibacterium*, and the presence of these did not reject true *Cutibacterium* infection (Supplementary Table 1, right column). True infection was also rejected if the patient had a focal infection caused by another pathogen (criterion 3). A focal infection was in turn defined by isolation of pathogens other than *Cutibacterium* at the site of infection in conjunction with either typical signs, symptoms, or imaging results compatible with focal infection. In episodes with only one blood culture positive for *Cutibacterium*, an additional criterion had to be fulfilled (criterion 4). For this criterion, the patient had to have either a foreign intravascular device present > 48 h prior to blood sample or *Cutibacterium* isolated at site of infection. Episodes of positive *Cutibacterium* blood cultures that did not meet these criteria were considered contaminations.

**Table 1:** Definition of true infection or contamination

Two or more positive blood cultures
*Criterion 1* Infection confirmed by one of the following at the time of the blood culture or within 48 h: a)Fever (temperature > = 38) b)Chills c)Leukocytosis (> 12 × 10^9^/L) OR Infection confirmed by the treating physicians at the discharge from the hospital
*Criterion 2* No other more likely pathogen in blood culture explains confirmed infection
*Criterion 3* No other focal infection with another pathogen can explain the symptoms of the patient where a focal infection is defined by: Isolation of pathogens other than *Cutibacterium* at the site of infection and one of the following: a)Typical signs or symptoms of focal infection b)Imaging results compatible with focal infection
Additional criteria in cases with one positive blood culture
*Criterion 4* Foreign intravascular device^a^ present > 48 h prior to blood sample OR *Cutibacterium* isolated at the site of infection

^a^Implantable cardioverters, pacemakers, prosthetic heart valves, central venous catheters, port-á-cath, intravascular grafts, and picc-lines. Stents placed after percutaneous coronary interventions and peripheral venous catheters were not included

### Statistical analysis

For categorial data, Pearson’s chi-squared test was performed for multiple comparisons. In cases where the sample size was small or for pairwise comparisons, we used Fisher’s exact test instead. For continuous variables, the non-parametric Mann–Whitney *U* test was performed. A *p*-value of < 0.05 was regarded as statistically significant.

## Results

### Description and characteristics of the cohort

**Fig. 1:**
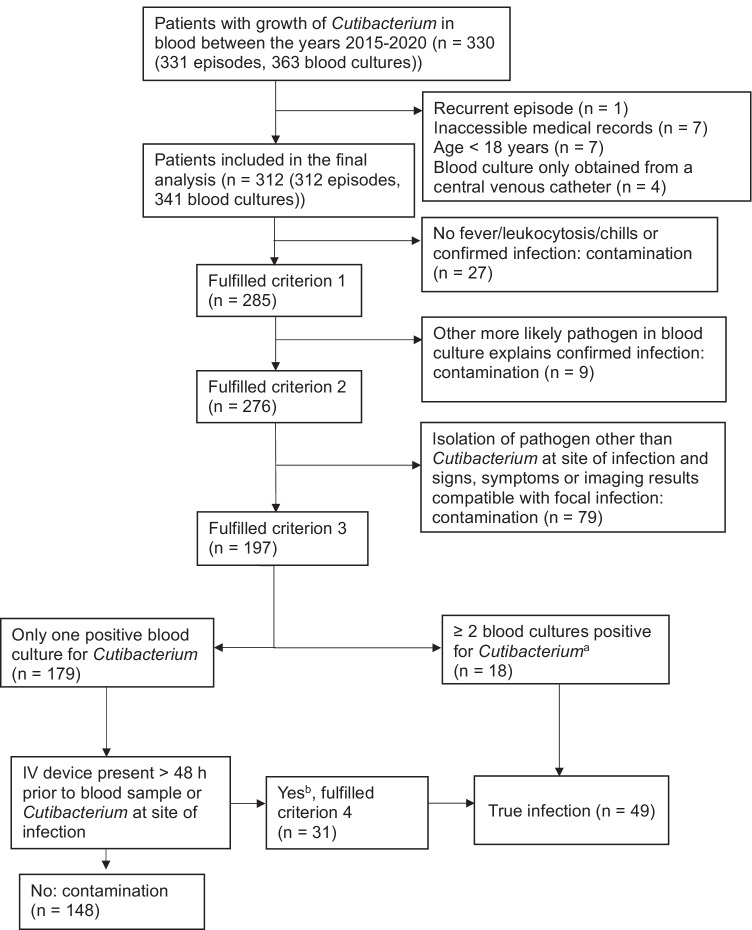
Flowchart of patients that fulfilled the criteria for true infection. ^a^Included one episode of Cutibacterium isolated at the site of infection and seven patients with an intravascular device. ^b^All patients had an IV device present, and in two episodes, Cutibacterium was isolated at the site of infection

Growth of *Cutibacterium* was identified in 363 blood cultures from 330 patients with 331 episodes of bacteremia, of whom 312 patients were left after removing those where the exclusion criteria were met. A flow chart of patients that were included in the study and those classified with true *Cutibacterium* bacteremia is shown in Fig. 1. True *Cutibacterium* infections were found in 49 patients (16%) and contaminations in 263 patients (84%). Characteristics of the patients are listed in Table 2. In both groups, the majority were elderly males, but the median age was higher in the true infection group (*p* = 0.021). The median Charlson score was one point higher in the true infection group compared to the contamination group (2 vs 1), and this difference was statistically significant (*p* = 0.043). Almost half of all patients in the true infection group had, except for fever and chills, no signs or symptoms suggesting focal infection at the time point when the blood culture was taken.

**Table 2:** Characteristics of patients with true *Cutibacterium* infection and contamination

	True infection (*n* = 49)	Contamination (*n* = 263)	*P*-value of difference^c^
Age, median (IQR^a^)	74 (65–83).^b^	69 (53–85)	0.021
Male gender, *n* (%)	34 (69)	155 (59)	0.17
Immunosuppressive therapy	7 (14)	26 (10)	0.32
Use of chemotherapy	10 (20)	14 (5.3)	< 0.001
CRP^d^ median (IQR)	64 (27–159)	65 (24–132)	0.74
SOFA score (IQR)	2 (0–12)	2 (0–3)	0.11
Leukocytosis	27 (55)	152 (58)	0.71
Fever (temp ≥ 38)	36 (74)	153 (58)	0.044
Chills	11 (22)	51 (19)	0.62
Charlson score, median (IQR)	2 (0–3)	1 (0–2)	0.043
Intravascular device	36 (74)	21 (8.0)	< 0.001
Site of acquisition			0.81
Nosocomial	7 (14)	31 (12)	
Healthcare associated	22 (45)	113 (43)	
Community	20 (41)	119 (45)	
Symptom^d^			
Respiratory tract	19 (39)	75 (29)	
Urinary tract	0	37 (14)	
Skin	2 (4.1)	28 (11)	
Abdominal	4 (8.2)	32 (12)	
No signs or symptoms	24 (49)	84 (32)	0.021
Other	0	7 (2.7)	
Intensive care	11 (22)	46 (18)	0.41
In hospital mortality	7 (14)	29 (11)	0.51
Death within 30 days	8 (16)	27 (10)	0.22
Death within 60 days	6 (12)	22 (8.4)	0.38

^a^Interquartile range. ^b^Episode numbers and percent. ^c^Fisher’s exact test, Pearson’s chi-squared, and Mann–Whitney *U* test were performed to compare the groups. ^d^At the time of the blood culture

### Microbiological features of the isolates

Species were determined in 281 patients (90%) and *C. acnes* was the most common species, found in 275 patients. Three patients each had isolates determined as *C. avidum* and *C. granulosum.* The microbiological features of the isolates are listed in Table 3. The most common species determined in isolates from patients with true infection was *C. acnes* (*n* = 44), a single isolate was *C. avidum*, and four isolates were only determined to the genus level. Only three patients had *Cutibacterium* isolated at the site of infection, all determined as *C. acnes*. The median time to blood culture positivity was significantly lower in the true infection group, 100 h (IQR 53–129) compared to 106 h (IQR 97–115) in the contamination group (*p* = 0.002). All but three tested isolates had a pattern of antimicrobial susceptibility typical for the genus, including resistance to metronidazole and sensitivity to penicillin, vancomycin, and clindamycin. These three isolates were resistant to clindamycin.

**Table 3:** The microbiological features of the isolates in patients with true infection and contamination

	Total (*n* = 312)	True infection (*n* = 49)	Contamination *(n* = 263)	*P* for difference^a^
Two or more positive blood cultures	29 (9.3).^b^	18 (37)	11 (4.2)	< 0.001
Polymicrobial	47 (15)	8 (16)	39 (15)	0.79
TTP^c^ (IQR.^d^)	105 (96–114)	100 (83–109)	106 (97–115)	0.002
Species of *Cutibacterium*, *n* (%)				
* C. acnes*	275 (88)	44 (90)	231 (88)	
* C. avidum*	3 (1.0)	1 (2.0)	2 (0.8)	
* C. granulosum*	3 (1.0)	0	3 (1.1)	
Not determined to species	31 (10)	4 (8.2)	27 (10)	

^a^Pearson’s chi-squared and Mann–Whitney *U* test was performed to compare the groups. ^b^Episode numbers and percent. ^c^Time to blood culture positivity in hours. ^d^Interquartile range

### Infections caused by Cutibacterium

The sites of focal infection are shown in Table 4. A plurality of patients with true *Cutibacterium* infection had an unknown focus of infection (43%). The most common known focus of infection was the respiratory tract (37%), such as pneumonia. Below, we describe five cases of severe infections caused by *Cutibacterium* (Supplementary Table 2)*.*

**Table 4:** The site of infection in patients with true infection

Focus of infection	True infection (*n* = 49)^a^
Respiratory tract	18 (37)
Urinary tract	3 (6.1)
Abdominal	1 (2.0)
Skin	1 (2.0)
Graft and stent	3 (6.1).^b^
Shunt	1 (2.0).^c^
IE	1 (2.0).^d^
Unknown	21 (43)

^a^Episode numbers and percent. ^b^Two *Cutibacterium* were isolated at the site of infection. ^c^*Cutibacterium* isolated at the site of infection. ^d^Had a biological aortic valve prosthesis

One patient was diagnosed with a VP-shunt infection at the relapse of *Cutibacterium* bacteremia. This was a 79-year-old woman with a VP-shunt, placed 6 years earlier due to idiopathic normal pressure hydrocephalus. Two blood cultures were positive for *Cutibacterium acnes.* The shunt was surgically removed, and cultures yielded growth for *C. acnes*.

Three patients in the cohort had infections of vascular stent-grafts, implanted after endovascular aneurysm repair procedures, and all were elderly men. One patient had two positive blood cultures for *C. acnes*, and from two patients, only one blood culture had been collected. Of the patients with only one positive blood culture for *C. acnes*, one underwent coil embolization, and one had their graft replaced. Tissue samples and cultures from the aneurysm were collected from these patients and yield growth of *C. acnes.* The patient with two positive blood cultures for *C. acnes* was not considered suitable for surgery, and therefore no culture from the aneurysm was collected. Instead, this patient was only put on lifelong amoxicillin treatment.

One patient in our cohort was diagnosed with IE. This was a 59-year-old man with a biological aortic prosthesis who had two positive blood cultures for *C. acnes.* The patient underwent a transesophageal echocardiogram that showed a 17 × 9 mm vegetation on the prosthetic aortic valve and high suspicion of an aortic root abscess. Thoracic surgery was therefore, later performed confirming IE. Cultures collected from the prosthetic aortic valve yielded no growth of bacteria, but the patient had been treated with antibiotics for 1 month before the surgery.

## Discussion

Our study indicates that patients with positive *Cutibacterium* blood cultures with true infection are relatively rare but that the finding of *Cutibacterium* in blood cultures should not always be considered contamination. We conclude the proportion of true infection in patients with positive *Cutibacterium* blood cultures to be 16% and the incidence of true bacteremia to be around six cases per million inhabitants per year. These numbers are higher than previous studies, which have presented a proportion of true infection in patients with *Cutibacterium* bacteremia between 0 and 3.5% [7–10]. One reason for this may be that in our definition, unlike previous studies, a patient with only one positive blood culture for *Cutibacterium* can still be considered to have a true infection. Two patients in our cohort with significant stent-graft infections, where *Cutibacterium* was isolated from the site of infection, had only one positive blood culture. This indicates that previous definitions of true *Cutibacterium* bacteremia, where all patients with only one blood culture positive were considered contaminations, may miss true infections. We used the same additional criterion for patients with only one positive blood culture as previously described by Rasmussen and co-workers in patients with positive *Corynebacterium* blood cultures. As *Cutibacterium*, *Corynebacterium* is a bacterium that also often contaminates blood cultures and in rare cases causes severe infections*.* The additional criterion that had to be fulfilled included a foreign intravascular device present or *Cutibacterium* isolated at the site of infection. Since the implantation of an IV device is becoming more common, this may be a reason for our presented proportion of true infection in patients with positive *Cutibacterium* blood cultures is higher than previous studies have demonstrated. Also, the study by Park HJ et al*.* [7] that presented a proportion of true infection of 3.5% only included *C. acnes* and thus not the other species within the genus.

Our definition of true *Cutibacterium* infection may increase the risk for false positives. True infection was rejected if the patient had a focal infection with another pathogen (criterion 3). A focal infection, in turn, necessitated the isolation of pathogens other than *Cutibacterium* at the site of infection. This will lead to a patient with pneumonia without any microbiological investigations or findings from the airways and with *Cutibacterium* in two blood cultures will be regarded as pneumonia caused by *Cutibacterium*. However, it is possible, and even plausible that the pneumonia was caused by another organism. In sputum samples, *Cutibacterium* will not be isolated or identified by the laboratory, making it impossible to confirm *Cutibacterium* etiology using a retrospective study approach.

Using our criteria, there is also a risk for false contaminations. For example, in some patients, only one blood culture was collected, and if these patients did not have an IV device present or *Cutibacterium* isolated from the site of infection (criterion 4), *Cutibacterium* was considered contamination. If two blood cultures would have been collected from these patients, it is possible that the other blood culture also would have yielded growth of *Cutibacterium* and, thus, considered true infections instead. Also, when cultures are collected from non-sterile sites, the growth of *Cutibacterium* is rarely recorded since *Cutibacterium* is a slow-growing anaerobic bacterium and is usually not considered a primary pathogen. Also, infections with *Cutibacterium* may have an insidious onset and remain unnoticed by the treating physicians. However, we only found one patient with recurrence of positive *Cutibacterium* blood cultures, and this might indicate that not many true infections were missed.

In line with previous studies, we report that *C. acnes* is the most common species within the genus to be found in isolates from patients with positive blood cultures with *Cutibacterium* [38]*.* In our study, there were too few episodes of positive *Cutibacterium* blood cultures with species other than *C. acnes* to draw any conclusions about their clinical features.

Our work demonstrates that a short time to blood culture positivity can be associated with true *Cutibacterium* infection, though the difference was too small to be used for clinical decision-making*.* This result is, however, in line with several studies, which have reported that time to blood culture positivity can be at use when distinguishing between contamination and true infection in patients with positive blood cultures for coagulase-negative *Staphylococci* and *Corynebacterium* [39–42].

The strengths of our study are that it is population-based and that the cohort is relatively large. Another strength is that the blood cultures were collected from the same laboratory and, therefore, the same methods for species determination were used for all isolates. However, this implies that all isolates included in the study are collected from a limited geographic area. Our study is, to the best of our knowledge, the only one to investigate the incidence, microbiological and clinical features associated with contamination and true infection in patients with positive *Cutibacterium* blood cultures since the reclassification of the genus. The limitation of our study is the retrospective design, leading to that only information about the patients noted in the medical records was available.

In conclusion, our study presents that patients with positive *Cutibacterium* blood cultures rarely have a true infection but that *Cutibacterium* should not always be considered contamination. Definitions of true *Cutibacterium* bacteremia that only consider the number of positive blood cultures may miss true infections.

## Supplementary Information

Below is the link to the electronic supplementary material.

Supplementary file1 (DOCX 16 KB)

Supplementary file2 (DOCX 17 KB)

## Data Availability

The datasets analyzed during the current study are not publicly available due to individual privacy but are available from the corresponding author on reasonable request.
