# Supplementary material for: True infection or contamination in patients with positive Cutibacterium blood cultures—a retrospective cohort study
Source: Eur J Clin Microbiol Infect Dis. 2022 May 25;41(7):1029–37. doi: 10.1007/s10096-022-04458-9 (PMC9250478; doi:10.1007/s10096-022-04458-9)
Supplement: Supplementary file 1 — Supplementary file1 (DOCX 16 KB) [file 10096_2022_4458_MOESM1_ESM.docx]

**Supplementary table 1.** Other bacteria in blood cultures with *Cutibacterium*

| **True infection rejected (n = 8)** ^a^ | **True infection not rejected (n = 39)** |
| --- | --- |
| *Escherichia coli* (2) | *Coagulase-negative Staphylococcus* (29) |
| *Citrobacter freundii* (1) | *Micrococcus species* (4) |
| *Fusobacterium necrophorum* (1) | *Anaerococcus species* (2) |
| *Haemophilus influenzae* (1) | *Corynebacterium* (2) |
| *Staphylococcus aureus* (1) | *Fusobacterium species* (1) |
| *Streptococcus anginosus* (1) | *Staphylococcus* *epidermidis* and *Rothia mucilaginosa* (1) |
| *Streptococcus dysgalacticae* (1) |  |

^a^ Episode numbers
