# Supplementary material for: True infection or contamination in patients with positive Cutibacterium blood cultures—a retrospective cohort study
Source: Eur J Clin Microbiol Infect Dis. 2022 May 25;41(7):1029–37. doi: 10.1007/s10096-022-04458-9 (PMC9250478; doi:10.1007/s10096-022-04458-9)
Supplement: Supplementary file 2 — Supplementary file2 (DOCX 17 KB) [file 10096_2022_4458_MOESM2_ESM.docx]

**Supplementary table 2.** The microbiological features of isolates and characteristics in five cases of severe infections caused by *Cutibacterium.*

| **Focus of infection** | **Gender** | **Age** | **Species** | **No. of positive blood cultures** | **Polymicrobial** | **TTP^a^** | **Outcome** |
| --- | --- | --- | --- | --- | --- | --- | --- |
| VP ^b^-shunt | Female | 79 | C. *acnes* | 2 | No | 104 | Relapsed ^c^ |
| Vascular stent-graft | Male | 80 | C. *acnes* | 1 | No | 114 | Cured |
| Vascular stent-graft | Male | 68 | C. *acnes* | 1 | Yes ^d^ | 55 | Cured |
| Vascular stent-graft | Male | 83 | C. *acnes* | 2 | No | 97 | Suppressive therapy |
| Infective endocarditis^e^ | Male | 59 | C. *acnes* | 2 | No | 107 | Cured |

^a^ Time to blood culture positivity in hours. ^b^ Ventriculo-peritoneal. ^c^ New episode after 83 days with positive blood cultures for *Cutibacterium.* At the relapse successfully cured with surgical shunt removal. ^d^ *Anaerococcus species.* ^e^ Had a biological aorthic valve prosthesis.
